# Young people in Australia discuss strategies for preventing the normalisation of gambling and reducing gambling harm

**DOI:** 10.1186/s12889-022-13201-0

**Published:** 2022-05-12

**Authors:** Hannah Pitt, Samantha L. Thomas, Melanie Randle, Sean Cowlishaw, Grace Arnot, Sylvia Kairouz, Mike Daube

**Affiliations:** 1 Faculty of Health, Institute for Health Transformation, Deakin University, Geelong, Australia; 2 Faculty of Business and Law, University of Wollongong, Wollongong, Australia; 3 Department of Psychiatry, University of Melbourne, Melbourne, Australia; 4 Institute for Health Transformation, Deakin University, Geelong, Australia; 5 Gambling Studies, Concordia University, Montreal, Canada; 6 Faculty of Health Sciences, Curtin University, Perth, Australia

**Keywords:** Young people, Children, Gambling, Gambling harm, Normalisation, Prevention

## Abstract

**Background:**

The normalisation of gambling for young people has received considerable recent attention in the public health literature, particularly given the proliferation of gambling marketing aligned with sport. A range of studies and reports into the health and wellbeing of young people have recommended that they should be consulted and engaged in developing public health policy and prevention strategies. There are, however, very few opportunities for young people to have a say about gambling issues, with little consideration of their voices in public health recommendations related to gambling. This study aimed to address this gap by documenting young people’s perceptions about strategies that could be used to counter the normalisation of gambling and prevent gambling related harm.

**Methods:**

This study took a critical qualitative inquiry approach, which acknowledges the role of power and social injustice in health issues. Qualitative interviews, using a constructivist approach, were conducted with 54 young people (11–17 years) in Australia. Reflexive thematic analysis was used to interpret the data.

**Results:**

Five overall strategies were constructed from the data. 1) Reducing the accessibility and availability of gambling products; 2) Changing gambling infrastructure to help reduce the risks associated with gambling engagement; 3) Untangling the relationship between gambling and sport; 4) Restrictions on advertising; and 5) Counter-framing in commercial messages about gambling.

**Conclusions:**

This study demonstrates that young people have important insights and provide recommendations for addressing factors that may contribute to the normalisation of gambling, including strategies to prevent gambling related harm. Young people hold similar views to public health experts about strategies aimed at de-normalising gambling in their local communities and have strong opinions about the need for gambling to be removed from sport.

## Background

Gambling is recognised as a global public health issue [1, 2], with the contemporary gambling landscape described as a ‘threat to public health’ [3]. While financial losses are most commonly described as the major harm associated with gambling, other negative health and social issues relating to gambling include mental health issues and stress, relationship breakdown, housing instability, family violence, crime, and energy poverty [4–6]. Australia has been described as having one of the most normalised gambling environments in the world, with over AUD 25 billon lost on gambling in 2018/19 [7]. A range of gambling products—from lotteries to sports betting and high intensity electronic gambling machines (EGMs or poker machines) —are embedded in everyday environments, alongside a range of innovative and omnipresent marketing strategies. As an example, with the exception of Western Australia, there are approximately 200,000 EGMs (often described as the most harmful gambling product), located within community clubs and hotels [8]. Gambling is mostly regulated by State and Territory governments, although some gambling matters such as online gambling and advertising are legislated by the Federal Government [9].

In the last decade, there have been a range of community concerns raised about the rapid expansion of new forms of gambling, such as online sports betting, the alignment of these forms of gambling and their marketing with valued cultural activities such as sport, and the potential impact on the normalisation of gambling for children and young people [10, 11]. Bunn and colleagues [11, pg. 827] state that while gambling companies have utilised the popularity of sport to increase the visibility of their products and brands, *“the relationship is deeper than this, however: gambling seems to have become entangled with the act of consuming sports…”*. The consumption of gambling products is generally only legal for those aged 18 years and older in Australia. However, a recent survey in New South Wales found that 29.9% of 12–17 year olds had gambled with money in the past year, with 1.5% classified as having problem gambling behaviours, and a further 2.2% at risk of problem gambling [12]. Another longitudinal study of Australian children found that 5% of 16–17 year olds had gambled with money in the past year, with 2.8% classified as having problem gambling behaviours, and 9.3% at risk of problem gambling [13].

In 2020, the WHO-UNICEF-Lancet Commission on ‘A future for the world’s children?’ stated that gambling is a “*potentially large and unaddressed public health challenge for children*” [14, pg. 631]. The Commission drew attention to research showing children's awareness of, and receptivity to, gambling advertising, products, and sponsorship. This research has increasingly focused on the processes that contribute to the normalisation of gambling in community and online settings, especially as they relate to young people [15, 16]. The primary focus of this literature has been on how children’s exposure to gambling marketing across multiple media channels may shape or influence their gambling attitudes and future consumption intentions [17–19]. This research has also demonstrated that gambling is already normalised for many children. By way of illustration, around 75% of young people in some studies in the United Kingdom and Australia agree that gambling is a normal or common part of sport, with many young people forming this opinion based on marketing aligned with sporting matches [17, 20].

Other studies have demonstrated that gambling products that are embedded in community settings, such as lotteries, or associated with family friendly venues, such as clubs and hotels (which host large numbers of EGMs in Australia), may contribute to young people’s perceptions that these forms of gambling are a normal part of everyday life [21–23]. This may be partly due to young people’s constant exposure to gambling products in community settings. For example, in a study involving young people aged 11 to 13 years using wearable cameras, Smith and colleagues [24] found that exposure to gambling marketing in New Zealand most commonly occurred in bookstores, convenience stores, and supermarkets, with lotteries and scratch cards the most frequently promoted products. Along with marketing, a range of traditions and social norms are also associated with the normalisation of gambling, including activities aligned or embedded within major social and cultural events such as birthdays, celebrations, and national holidays [25, 26].

Many of the aforementioned studies have recommended that young people should be consulted and engaged in developing prevention and public health policy responses to gambling. Some limited conversations about gambling may occur with young people via school-based education programs developed and delivered by adults, which aim to help students “*navigate the new gambling landscape*” and “*avoid harm from gambling*” [27]. There are, however, very few opportunities for young people to have a say about gambling issues, including to politicians and policy makers. While there is increasing momentum to empower young people to have a say in decision making processes related to some salient issues, such as climate change [28, 29], there is very little consideration of young people’s voices in public health recommendations as they relate to gambling.

While a number of studies have examined the extent to which adults support strategies and policies aimed at preventing gambling harm [30–32], there are few studies that have asked young people what they think could be done to address the normalisation of gambling. Some preliminary studies have focused on young people’s responses to gambling advertising, and suggest that the majority believe that there should be less or no gambling advertising on television, and that sporting codes should do more to protect young people from exposure to advertising [16, 19, 33]. Young people also perceive that educational strategies such as school based education and campaigns may have a role to play as part of a comprehensive public health approach to gambling [16, 33]. Torrance et al. [34] found that 18 to 29 year olds perceived that ‘responsible gambling’ messages were largely tokenistic and ineffective, that industry was doing the bare minimum to reduce harm, and that stricter regulations associated with advertising were needed [pg. 8]. However, few studies have asked young people to consider a broad range of strategies that may be used to prevent the normalisation and harms associated with a range of different types of gambling. The present study aimed to fill this gap in knowledge, and was guided by three research questions:

- 1. What are the strategies that young people perceive are useful to prevent the normalisation of gambling and gambling harm?
- 2. Are young people’s views consistent with broader population public health measures intended to prevent the normalisation of gambling and gambling harm?
- 3. How can the perspectives of young people be better incorporated in decision making about strategies to prevent the normalisation of gambling and gambling harm?

## Methods

### Approach

The data in this paper was part of a broader study investigating the normalisation of gambling for young people in Australia. In interpreting the data, the researchers took a public health approach to data interpretation, which acknowledges that gambling practices and potential harms are driven by a range of determinants, including social and environmental contexts, and the influence of the gambling industry and regulatory frameworks [35]. The study took a critical qualitative inquiry approach which acknowledges the role of power and social injustice in health issues and aims to use study findings to inform social and policy change around a particular issue [36–38]. This approach was chosen based on expert commentaries from public health researchers, who have argued that approaches to gambling policy must be guided by *“principles of health, equity, and social justice”,* and *“shaped by democratic processes that welcome public voices…”* [3, pg. e614]. This critical qualitative inquiry approach guided all aspects of the study, including the development of implications for public health approaches to policy and practice.

### Sampling and recruitment

Young people aged 11 to 17 years were invited to participate in the study through their parents or primary carers. This age range was chosen assuming that this is the age at which many young people start to think about and become aware of gambling, and are able to critically interact with the information that they see about gambling [18, 39]. A range of convenience, purposive, and snowball techniques were used to invite participation in the study. These included distributing recruitment notices on social media sites (for example, posting the flyer to Twitter and Facebook), contacting parents through our existing networks, and asking parents to pass on information about the study to other parents and families. Purposive sampling strategies were used to ensure that young people with a wide range of attitudes towards gambling were invited to participate [40]. Recognising the influence of social contexts on gambling attitudes and behaviours, this study sought to recruit young people from different socio-demographic and geographic contexts. Parents were provided with a Plain Language Statement about the study and were asked to share details with their child. Written consent was obtained from parents. Interviews were conducted via videoconference, and initially involved researchers revisiting the main points of the Plain Language Statement and the consent process. Young people were then invited to ask questions about the study before providing verbal consent. Participants were told that there were no right or wrong answers, that they could slow down or stop the interview at any time, and that the research team were interested in their attitudes and opinions. Young people were provided with a AUD 30 grocery voucher as a token of appreciation for their time. Approval for the study was received from the Deakin University Human Research Ethics Committee [2019–534].

### Data collection

Semi-structured interviews, lasting approximately one hour, were conducted between July 2020 and April 2021 via videoconference (due to social distancing restrictions associated with the COVID-19 pandemic). Young people were able to participate in the interview by themselves, or with siblings who were also between 11–17 years if they felt more comfortable doing so. Interviews were audio-recorded with permission, and were professionally transcribed. Transcripts were read by members of the team, and the recording was revisited if there were questions or clarifications needed about the accuracy of the transcription.

The broader interview included questions that related to young people’s social media use, sports viewing, recall and awareness of gambling advertising, gambling intentions, the normalisation of gambling, and harm reduction strategies. In relation to this study, young people were asked to reflect on strategies that could be used to counter the normalisation of gambling or reduce gambling related harm. Given that gambling is sometimes a complex issue for young people to reflect upon, and that most research to date has focused mainly on online forms of gambling, a range of visuals were used to prompt young people’s thoughts and opinions about gambling. For example, young people were shown pictures of various gambling products, as well as infographics relating to the amount of money spent on gambling advertising in Australia. Leonard and McKnight [41] argue that these types of visual methods are an important tool in research with young people as they “…*encourage more collaborative research by reducing power imbalances in the research process and can help in tapping into experiences that are not easily conveyed or captured verbally, issues that can be particularly relevant in research with young people*” [pg. 629]. To help young people think about policy issues and recommendations, they were asked about what they would say about gambling to politicians or to sporting organisations. For example, “*what would be your message to the sporting codes, teams, and athletes about gambling and sport?*” and “*what sorts of things do you think could be done to prevent the risks of gambling in our communities?*” Data collection was discontinued when it was determined that the depth of data collected across the interviews provided enough ‘information power’ to elucidate the broad overall aims of the study [42].

### Data interpretation

Data interpretation was guided by a constructivist paradigm, exploring how young people made sense of gambling environments [43]. Braun and Clarke’s six steps of reflexive thematic analysis were utilised as an inductive, iterative process of data interpretation [44, 45]. In step one, members of the team became familiar with the data through reading and re-reading transcripts, noting ideas and thoughts about how young people conceptualised how and why gambling was normalised, and perceptions of how harms associated with gambling could be reduced. As interviews were read as part of step two, codes were generated about different aspects of gambling normalisation and harm reduction strategies, particularly as they were associated with things that young people had seen in their everyday lives. Themes were then constructed from the data (step three). These themes were reviewed by members of the research team (step four), and further refined to reflect key harm reduction strategies and address the research questions (step five). Findings were finalised during the write up of the manuscript (step six). During this process, the researchers reflected on the core tenets of critical qualitative inquiry, including any issues that emerged from the data relating to power and/or social justice. This was particularly important in light of descriptions of the responsibilities of powerful social agencies and corporations, such as sporting organisations, governments, and the gambling industry.

To ensure reflexivity, members of the team met regularly to consider and discuss the main themes that were constructed from the data. This included how subthemes and themes could be explained by the broader research literature, and new areas for consideration. For example, while on the surface young people presented clear strategies for reducing gambling harm, there were also a range of underlying themes relating to young people’s social consciousness and empathy for people who had experienced problems with gambling. This process was also used in the development of the model to emerge from the data, with regular written and in person feedback loops enabling the research team to provide comment and reflection. The quotes presented in this paper are used to enhance the trustworthiness of the data, illustrate key categories, and importantly, to ensure that young people’s voices were clearly represented in the presentation of the results [46].

## Results

### Sample characteristics

A total of *n* = 54 young people from 36 families participated in the study. Individual interviews were held with *n* = 43 young people, and five interviews were conducted with groups of siblings. The sample was relatively evenly distributed by gender (*n* = 25 girls and *n* = 29 boys), and was slightly skewed towards younger participants (*n* = 34, 11–13 year olds and *n* = 20, 14–17 year olds). The majority of young people were residents in the state of Victoria (*n* = 40), with six participants from Queensland, five from New South Wales, and three from the Northern Territory.

Five themes were constructed from the data.

### Theme one: Reducing the accessibility and availability of gambling products

Young people offered suggestions about strategies that could be implemented to reduce the availability and accessibility of gambling products. Most of these related to community rather than online settings – for example EGMs and betting shops (or Totalisator Agency Board (TABs)). Initial reactions from young people were strong – and included recommendations to completely ban some forms of gambling, such as EGMs. For example, some young people stated that EGMs needed to be closed, banned, or temporarily restricted at certain times of the week or year to give people a break from gambling. Others suggested getting rid of EGMs, or that people should be stopped from using them:

Stop it. Close down all the poker machines. Just try and limit the amount of people that can gamble at all or at one time because gambling isn't cool. - 12-year-old male, Queensland

I feel like we should reduce, like, pokies machines, or have – yeah, just reduce them, or get rid of them from some venues. - 13 year-old-female, Victoria

Young people perceived that these restrictions would ensure that individuals were not able to spend as much money on certain forms of gambling, particularly EGMs. Suggestions included changing gambling environments to ensure that they were less appealing for individuals to want to stay in for a long time. Some others focused on more targeted approaches, such as removing gambling from areas that were popular social spaces or contained other risky products (such as alcohol), to make gambling less of a normal part of everyday life. One young person tried to illustrate this by stating that gambling venues should only be in places where tourists went:

I think maybe removing them from local bars and things, I guess could help. And maybe putting them only in certain places where a lot of tourists may go. So it's not a thing that you can do every weekend, I guess. That it's only a fun thing to do when you're on a holiday, or something like that? - 12- year-old-male, Victoria

While some young people recognised that gambling would always be part of social group activities, they perceived that restrictions would ensure that gambling was not an activity that was easily accessible in communities or online spaces:

Yeah, I think they should have less [gambling] and they should have less pokies venues and they should have less machines. They should limit the amount of people in the pokies rooms… The pokies venues still get a lot of money it’s just they don’t get as much because people have to wait their turn, and they’re not allowed that much people in there. - 13-year-old male, Victoria

### Theme two: Changing gambling infrastructure to help reduce the risks associated with product engagement

Young people recommended several changes to the structural characteristics of gambling products, and the infrastructure that surrounded these products. These proposed changes mostly included limiting the amount of time and money that individuals were able to spend on gambling. Young people regularly drew on their own observations of gambling environments in developing these recommendations. For example, young people were aware that individuals often spent many hours in venues, or lost track of the time that they spent on gambling:

Like sometimes when I’ve gone to our friend’s farm one time we stopped by this old pub, and there was like this room for pokies. There was just some guys that were just sitting there and they just seemed like they were sitting there all day just doing it [gambling]. …. I just feel like some people would be sitting on that all day, they might end up leaving the place with zero dollars because they’ve lost so much….. - 12-year-old male, Queensland

However, young people also recognised that EGM venues would not voluntarily implement restrictions that would decrease the time people spent gambling. The following 14 year old noted that government regulation was needed to ensure that individuals were not sitting in a venue for lengthy periods losing money:

Obviously, pokies are not going to make it easier for people to win. Pokies, they want – the more you play, the better they do, the more money they make…..I feel like the government, if they have the power to do something, they should make something where you can only be inside a pokies venue for a certain amount of time… You don’t want pokies venues, but if they’re still going to be open, then make people not have the power to just sit there and just spend their money. ..... There should be a limit to: 1), the amount of time you can spend in a venue, and 2), the amount of money that you can actually put in….. Then, I feel like after maybe two hours, I think they should kick you out of the place. You should not be able to be in a venue for more than two hours…, I think the venue should be encouraged to just not let them keep losing their money. - 14-year-old male, Victoria

The second major proposed change to gambling infrastructure related to caps on the amount of money that individuals could spend gambling. Young people recommended various strategies that could help individuals to monitor and avoid gambling losses, including providing links to direct information about how much money was left in their bank account, clocks to show time spent gambling, spend indicators, and tools to help individuals pre-commit to the money that they wanted to spend when gambling. The most common suggestions were caps on spending, including weekly caps, and legal limits to how much people were allowed to gamble in one session:

I would say put a cap, a limit for people and make it kind of lower, so under $100 a day for each person or you can't do it - the weekly cap is $500 or something like that and even that would still be quite a lot. Just for the extreme gamblers so they don't feel outraged or anything. Sort of still reasonable. - 14-year-old male, Victoria

Yeah. I think that we should, like, have a certain amount – like, a daily cap, or maybe a monthly cap, on how much a person can spend on – probably monthly – because that’s just, like, a lot that’s being used, and probably wasted in a way, as well. – 15 -year-old female, Victoria

Young people often thought through different scenarios when recommending these strategies, including considering how people might be able to work around restrictions, as well as mechanisms to stop that from happening:

I don't know how you'd manage it, but one way would just to be... put a limit on how much they can spend per night. I don't know, if they did it, they’re just going to rock up to the next pub, they won’t come back to your pub, but maybe if they just can communicate, or I don’t know, between local pubs, saying "Joel is here tonight, he spent this much money”. Or whatever. So a restriction on how much someone can actually put in per week. - 15-year-old female, New South Wales

### Theme three: Untangling the relationship between gambling and sport

Many young people discussed the pervasive and embedded nature of gambling within Australian sport, and referred almost exclusively to sports betting in this context. Some stated that the alignment between sport and harmful products such as gambling was problematic for individuals who were fans of sport because it was considered extremely popular and highly influential on the gambling decisions of some sports fans. Some young people had negative opinions about the relationship between gambling and sport, and the advertising of gambling in sport:

I’m a bit disappointed and sad that gambling is such a big part of sport now. I would say that like just do it because, watch it and do it because you love [sport] and don’t try to bring gambling into it. It doesn’t have to be about that, it doesn’t have to be about money. - 14-year-old female, Victoria

Some participants were critical about how normalised gambling had become within sport, and that individuals felt that they needed to gamble to be part of sport and sporting culture. They stated that stronger efforts should be made to get sport back to its core values of participation and enjoyment:

I don’t think you should gamble on sport because sport isn’t made for gambling, sport’s made for people to play sport. [It’s for people to] watch and enjoy, and people to play and enjoy, rather than spending money on it. - 16-year-old male, Victoria

A few young people stated that when sporting organisations endorsed gambling, they were simply encouraging gambling companies to make more money and to continue to advertise their products. The following young person commented on sponsor relationships between gambling companies and sporting organisations:

Well, again, it is their choice but I think there should be less of a presence of gambling companies sponsorship in sports because there’s – like why do they need more money to fuel them to keep advertising? - 11-year-old male, Victoria

A few young people stated that the relationship between gambling and sport needed to end because gambling put too much pressure on athletes from fans. This included that there was more pressure for athletes to perform if people had bets on the outcomes of a match, and that the relationship between gambling and sport could create pressures on athletes and teams:

I think it's okay, but I can see how people would see it as a bad thing. Especially if you're a team member, to have that pressure on you if people that are putting their money on your performance or your team's performance. I think that's a lot of pressure on the teams and the players themselves, because if they don't live up to that performance, then the people that support them are losing their money. - 14-year-old female, New South Wales

Participants also discussed the responsibility of sporting organisations to take a stand on gambling. Some stated that it was important for such organisations to be responsible towards their fans, and not be so reliant on the money they receive from companies that caused harm. Some stated that sporting organisations should recognise and reflect upon the extent of gambling harm in communities, considering their relationships with the gambling industry rather than *“just doing it for the money”.* Young people spoke extensively about the power of sporting organisations to promote gambling, but also about their power to prevent gambling harm. For example, some young people presented clear messages that sporting organisations should not “*promote*” or “*enforce*” gambling, with one stating that sporting organisations and athletes should not *“represent yourself through a betting thing”.* However, there was a perception that sporting organisations and athletes could play a significant role in shifting social norms in relation to gambling, and in encouraging individuals to not gamble:

Can you please just say one day in your post-match speech “don't gamble, please”. Because I bet [athletes] would have a huge amount of influence… That'd probably make a lot of people not gamble. So, probably if they talked about gambling and told everybody not to gamble, then that would be a huge influence across the country and there would be a lot less people gambling. - 12-year-old male, Queensland

While some young people stated that gambling should be removed from sport, there was a degree of scepticism that this would ever happen. It was perceived that the financial benefits to sporting organisations from gambling would make any decrease in gambling within sport difficult to achieve:

I don't like gambling in sport. I think it should be left out. It's one of those things that's been around for too long. It probably won't be stopped… It builds up the competition and the AFL [Australian Football League] or the sporting things might promote it, so they can get more people into it and pay more money. - 14-year-old male, Victoria

A few young people believed that the relationship between gambling and sport was positive. These participants stated that significant financial benefits for teams were built from sponsor relationships with the gambling industry:

I feel like it does belong in the sporting industry....I feel like it has a benefit on the sporting industry as well, because once you’ve gambled, you’re kind of forced, in a way, to watch the match, or the sport, so it could give more of a boost to that sport or something. - 13-year-old female, Victoria

### Theme four: Restrictions on advertising

Young people gave an overwhelmingly clear message that there should be much less or no gambling advertising. They felt that such advertising was particularly influential in both normalising and influencing individuals to gamble. They recommended strong curbs on gambling marketing, with suggestions ranging from limiting the number of and spend on gambling advertisements, to complete bans. When recommending limits on advertising, young people often expressed significant care and empathy for those who could be potentially harmed by gambling. Young people perceived that government, broadcasters, and the gambling industry were most responsible for reducing young people’s exposure to gambling marketing:

But yeah, I think the government is responsible, the television programs are responsible. Maybe, as well, the companies themselves. I think they should definitely be responsible with things like Instagram, like having Sportsbet ads – like, a lot of young kids use Instagram, like maybe I’d say 13 to roughly – 13 to 30-year-olds, maybe, maybe 13 to 25, something like that, but there’s a large young demographic that are really easy to grasp. - 12-year-old female, Queensland

While some young people perceived that gambling companies had a moral obligation to think about the harm that they were causing through advertising, particularly to young people, others commented that the gambling industry should not be involved in regulating their own advertising. This was because they perceived that their financial interests meant that they would not restrict their marketing in a way that would jeopardise profit. For example, one young person stated that it was the gambling companies’ job to make money from advertising and so it should not fall back on them to restrict their own marketing. Some young people recognised that restricting or banning advertising was a very complex process, which led them to be sceptical that advertising restrictions would occur:

If there's anything we can do, I suggest we at least limit the ads…. So many people are just getting snared in gambling every day. It's just not cool. I don't find it very cool. I feel like we should do that but I don't know anybody who would do it because it's difficult. It requires lawsuits and everything and there'd probably be a law case about it and we'd be crammed up for weeks, even with the crisis of coronavirus and I don't think it would be the best option to take right now. But I hope in the future someday, we'll be able to limit the amount of gambling ads. - 12-year-old male, Queensland

Others were sceptical about the willingness of government to regulate advertising, saying that governments made taxes from gambling and ultimately were as responsible as the gambling companies for harm:

I think that the actual companies and probably the government I reckon. Because honestly they are all about the money and the people who put the ads out there are the people that actually end up getting the money. It’s not like random people advertising it. So I reckon the people that should be held accountable and people who are responsible for it are the people like companies like Sportsbet and Crown Casino and the government. It’s like I reckon the government is trying to also play like the victim here, because they make it compulsorily to put in the line like “gamble responsibly” but at the end of the day they are getting taxes from it, .... they probably benefit from it in some sort of way. So the government and the actual companies who get it, like the house, kind of are responsible for these numbers. - 14-year-old female, Victoria

### Theme five: The need for counter-framing in commercial messages about gambling

Some young people were highly sceptical and critical of current harm prevention messages about gambling. Some had unprompted recall of responsible gambling messages that they had heard at the end of gambling advertisements. They often commented that gambling companies were forced to run the message, but they did not necessarily want to provide an effective harm prevention message. For example, one participant stated that the ‘gamble responsibly’ message was like an asterisk symbol which absolved the industry from responsibility and reinforced that it was the individual’s fault if they experienced harm from gambling:

Yeah like the gamble responsibly thing sort of at the very end when they're speed talking through the stuff that they have to put in the ads, it's the little asterisk thingies, but it's like yeah sort of I forgot how they put it, but it's like it's your fault if you lose all your money, gamble responsibly. …. It's like we are not responsible for any losses and stuff like that. - 14-year-old male, Victoria

Others noted the contradictions that were evident from having a ‘gamble responsibly’ message in commercial gambling advertisements. For example, one young person said that they thought that these messages were strange because on the one hand the gambling company was seemingly persuading individuals to gamble, and on the other hand the government was telling individuals not to gamble:

The only thing that I really remember is the gambling, like the stay safe while gambling from the government after because I always used to think oh well that’s kind of dissing their business because it’s like ‘oh come gamble here, but then also it’s bad to gamble’. That’s the thing that’s always comes up after the gambling ads from the government I think. - 12-year-old female, Victoria

While young people recognised that responsible gambling messages were intended to encourage individuals to be safe and in control with their gambling, few thought that this was an effective way of achieving this. Some stated that having short ‘gamble responsibly’ messages at the end of gambling advertisements was ineffective because they were less noticeable than the positive messages about gambling. They noted that the messages were in tiny writing, brief, and were said with speed:

Like at the end of each ad, they show the good part of the ad, at the end of the ad the screen just goes blank and goes “gamble responsibly” like stupid fast. Yeah, and there’s not really any control, no one can really process all of it. And you know, you can see these ads and you take in all the different stuff and then you are thinking about the good stuff and then like someone just sort of gives you a very quick warning and you don’t really have any time to process that. So they need to acknowledge like what can happen and yeah it’s smart, it’s what it is, it’s smart for the company, but for the public it’s not that helpful. - 16-year-old male, Victoria

While young people recommended that the best way to reduce harm and de-normalise gambling was to have strong curbs on advertising, they also recommended that messages aimed at counter-framing commercial gambling messages should be more prominent. They stated that these types of messages should be one of the main points in gambling advertising. Young people proposed a range of ways that harm prevention messages could be communicated about gambling. Some advocated that strong warning messages needed to be given about the risks associated with gambling, rather than focusing on responsibility. For example, one suggested that instead of governments running advertisements that encouraged individuals to gamble responsibly, they should run messages that said, “*don’t gamble*”. Others stated that there needed to be an emphasis on the extent and nature of gambling harm. This included showing statistics to ensure that individuals had a realistic understanding of how harmful gambling could be and creating awareness and showing the risks associated with gambling. Some advocated for strong and hard-hitting public health campaigns, similar to those used in tobacco that showed the direct harms of the product, and the impact it had on individuals who had been harmed by gambling:

I think like when the government put like the photos of like how bad it can be on cigarette packets……I don’t know how exactly they could do it but they should probably do something about that for gambling. Maybe like a flash ad or like on the screen of the pokies machine, like a flash ad kind of thing, like or showing someone who’s turned out bad because of it. - 12-year-old male, Queensland

A number of participants said that such messages should be targeted to young people and should try to steer young people away from gambling, rather than educating them to be responsible with the product. One participant stated that it was important that young people received these messages before they started to experiment with gambling, commenting that when individuals had no direct experience with gambling, they were potentially more vulnerable to negative outcomes from gambling products:

I feel like they might target young people ‘cause they're a bit vulnerable, we're coming in and learning, try and find our place in the world kind of thing. So maybe they should focus on showing the real thing rather than the kind of encouraging us to do it, which is obviously what they want to do to promote their company. But it's a bit harsh there's people that don't really know what they're doing. - 17-year-old female, New South Wales

A few young people stated that countering commercial messages about gambling should also involve discouraging gambling, and making gambling seem less exciting and fun. They said that this was important because the only messages that young people had seen were that gambling was an exciting, fun, and normal activity:

I think that from a young age, like, maybe even if the school did a program that teaches kids about the risk of [gambling]. ….If people learnt about the risk of it then they’ll be less likely to actually gamble. So I feel like kids, from a young age, should be taught that it’s not a good – I don’t know what to say because some people think it’s a good thing but I don’t think that it is because I can see you can lose a lot of money. So I think that kids should be taught the risks of it. Yeah, I think it is because they’re making it seem all exciting so kids will look forward to, like, be doing it when they’re older. But if schools teach you the risks of doing it, then they’re not going to think it’s as exciting. - 14-year-old female, Victoria

As one young person concluded:

Like, yeah, like tell everyone about the effects of it. If there were as many ads showing people that have had their lives stuffed up because of gambling, as there were ads telling you to do gambling, then I feel like it would be a much less harmful place. - 14-year-old male, Victoria

## Discussion

**Fig. 1:**
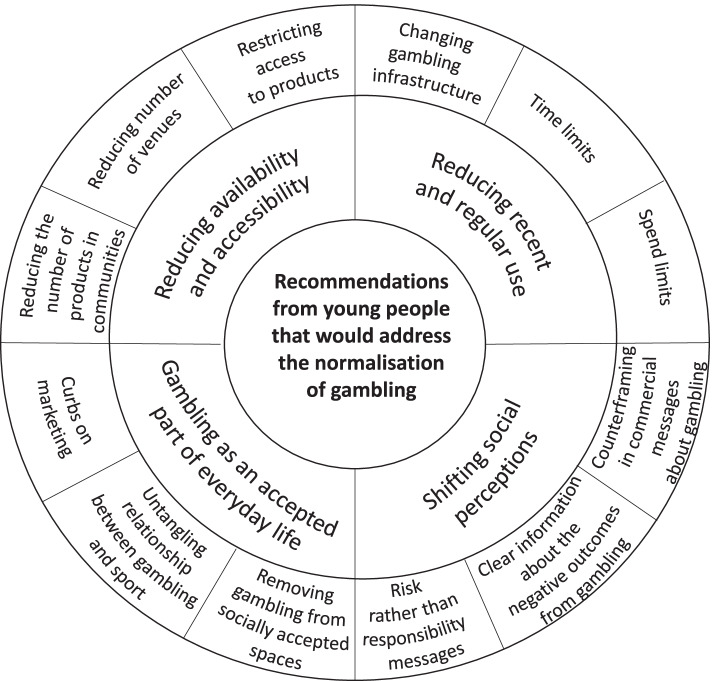
Strategies recommended by young people to address the normalisation of gambling and gambling harm

This study aimed to explore the strategies that young people perceived would be helpful in countering the normalisation of gambling, and in reducing gambling related harm. The key strategies suggested by young people included: 1) Reducing the accessibility and availability of gambling products; 2) Changing the nature of gambling products, and gambling infrastructure to help reduce the risks associated with gambling engagement; 3) Untangling the relationship between gambling and sport; 4) Restrictions on advertising; and 5) Counter-framing in commercial messages about gambling. These strategies have been mapped against the concept of normalisation and have been visually depicted in Fig. 1.

The findings from this study demonstrate that young people are capable of discussing strategies that could reduce the normalisation of gambling and prevent gambling related harm. It is important to recognise that strategies recommended by young people in this study are similar to de-normalisation and harm prevention strategies that have been endorsed by public health experts, key stakeholders, and those with lived experience of gambling harm [47–49]. For example, there is clear support within the public health community for reducing the accessibility and availability of gambling products in communities, specifically a reduction of EGM licences and gambling venue opening hours [31, 48]. There have also been many calls for increased regulation of gambling marketing, promotions, and sponsorships [14, 33, 48], whichmay normalise and encouragegambling among young people [16, 21]. The findings from this research demonstrate that young people not only have an important public voice in the discussion about public health responses to gambling related harm but are able to draw upon different types of evidence (including their own lived experiences) in providing recommendations.

To date, most research with young people has focused on factors that may contribute to the normalisation of gambling for young people. Young people have described different determinants that may contribute to the socio-cultural acceptability of gambling, including peer and family behaviours [21, 50], the embedding of gambling in culturally valued activities such as sport [16], and the impact and influence of excessive marketing across multiple media channels [19]. The present study demonstrates that young people are able to think about, contextualise, and suggest strategies that may help to counter the normalisation of gambling, even when they have not directly participated in gambling. The approaches that they proposed, associated with restricting the accessibility and availability of gambling products, changes to how individuals are able to interact with gambling products, curbs on marketing, and educational strategies associated with risk rather than responsibility, align with major public health and government reports in this area [9, 15]. This demonstrates that the concerns about the impacts of gambling products and their appeal are not limited to adults [48], but also come from young people.

Importantly, young people were able to consider how gambling may impact on people who may be vulnerable to harm and provide thoughtful and empathic responses about changes to the structural characteristics of gambling products and environments that would enable individuals to engage in gambling in a potentially less risky way. For example, young people thought about the pressures that sports betting may place on athletes from fans, and the impact of gambling on people who were vulnerable to losing track of time in venues. Similarly to studies in other areas of addiction [51], the present study demonstrates that young people were able to consider the social-cultural, environmental and political contexts in discussing strategies to reduce gambling related harm. This includes the perceptions that there should be restrictions on gambling marketing in sport [16, 33, 34], that sporting organisations should take responsibility for ensuring that they do not promote positive messages about gambling, and that governments should place curbs on advertising. However, it should also be noted that some young people were sceptical that the relationship between gambling and sport would change, because of the financial influences of the gambling industry, and potential implications for the sports involved. Bunn and colleagues [11] have previously highlighted that public health perspectives on gambling cannot be focused just on individual determinants, and must instead draw upon people’s experiences of their broader social contexts. While there is a tendency in gambling research to survey individuals about their behaviours, this study demonstrates that asking young people to reflect on their own gambling contexts and environments, rather than on their own individual behaviours, is an important mechanism for stimulating discussion about a range of strategies that could be used to reduce and prevent gambling related harm.

Young people were also able to critically reflect on current gambling harm reduction strategies, advocating for a range of educational strategies that provided clear and honest information about the negative outcomes associated with gambling. While previous research has demonstrated that young people are positive about the need for educational strategies [33], the present study provides more detail about the types of public messaging campaigns that young people perceive would be most influential. Although gambling education strategies have typically focused on individual and social determinants, young people considered a broad range of determinants that may contribute to gambling normalisation and harm. Young people were not convinced that the current framing of messages was effective. This particularly related to ‘responsible gambling’ messages that were perceived as disingenuous attempts to minimise harm within messages that promoted products. Rather, young people perceived the need for proper regulatory controls on industry to de-normalise gambling and prevent harm. Public health researchers and social scientists have repeatedly criticised ‘responsible gambling’ and personal responsibility paradigms [11, 52]. The results from this study with young people provide further evidence to support these criticisms, and for the development of independent (i.e., not funded, developed or delivered by industry) public education campaigns (including school based education programs) that aim to provide honest information to communities about the risks associated with gambling products [52].

It is important to understand young people’s perspectives and experiences about gambling and to ensure that they are provided an opportunity for their voices to be heard. The WHO-UNICEF-Lancet Commission emphasises that children currently have “*little voice in the shape of their future*” [14, pg. 607]. Young people have clearly demonstrated in other areas of public health that they are able to engage in discussions about policy issues such as alcohol and drug use, can recommend prevention strategies, and show willingness to engage in policy discussions and deliberations [53]. Research has also shown young people’s capacity to engage in critical feedback on suggestions about population based prevention activities that may impact directly on their age group [54]. There is an increasing focus in Australia on accessing young people’s views as a way of empowering them in relation to their health and wellbeing, and also in developing organisational cultures that are *“consultative, collaborative, and open to feedback and improvement”* [55, pg. 3]*.* The present study lends further support to engagement strategies that empower young people to contribute their perspectives about gambling in a meaningful way [33]. Developing authentic youth engagement and empowerment strategies will be essential in developing robust public health responses to the new challenges posed by gambling, and in utilising young people’s voices to drive action [56].

## Conclusions

Young people are able to consider and provide recommendations for ways of addressing factors that may contribute to the normalisation of gambling, including strategies to prevent gambling related harm. The present study demonstrates that young people are able to provide nuanced opinions and suggestions that move beyond simplistic messages to ban products and marketing. Young people hold similar views to public health experts about strategies aimed at de-normalising gambling in their local communities and have strong opinions about the need for gambling to be removed from sport. They are clear that government has a strong role to play in ensuring that individuals are protected from the harms associated with gambling. 

## Data Availability

The dataset analysed in the current study is not publicly available, or available on reasonable request because participants explicitly consented to only have their data shared with the immediate research team.
